# STAT3 is critical to promote inflammatory cytokines and RANKL expression in inflammatory arthritis

**DOI:** 10.1186/ar3644

**Published:** 2012-02-09

**Authors:** Takeshi Miyamoto, Tomoaki Mori, Akihiko Yoshimura, Toshiaki Toyama

**Affiliations:** 1 Department of Orthopedic Surgery, Keio University School of Medicine, Shinjuku, Tokyo, 160-8582, Japan; 2 Department of Immunology, Keio University School of Medicine, Shinjuku, Tokyo, 160-8582, Japan

## 

Rheumatoid arthritis (RA) causes sever joint damage and significant disability of daily living. The symptoms of RA patients are mainly from chronic inflammation and continuous joint destruction, however, the mechanisms underlying how inflammation and joint destruction in RA develop and are sustained chronically remain largely unclear. In this study, we show that signal transducer and activator of transcription 3 (STAT3) plays a critical role in both chronic inflammation and joint destruction in RA. We found that inflammatory cytokines, such as IL-1β, TNFα and IL-6, activated STAT3 either directly or indirectly and induced expression of inflammatory cytokines, further activating STAT3. STAT3 activation also induced expression of receptor activator of nuclear factor kappa B ligand (RANKL), an essential cytokine for osteoclast differentiation. STAT3 knockout or pharmacological inhibition resulted in significant reduction of the expression of both inflammatory cytokines and RANKL *in vitro*. STAT3 inhibition was also effective in treating an RA model, collagen induced arthritis (CIA), *in vivo *through significant reduction in expression of inflammatory cytokines and RANKL, inhibiting both inflammation and joint destruction. Thus our data provide new insight into pathogenesis of RA and provide evidence that inflammatory cytokines induce a cytokine amplification loop via STAT3 that promotes sustained inflammation and joint destruction.

